# Safeguarding Health at the Workplace: A Study of Work Engagement, Authenticity and Subjective Wellbeing among Religious Workers

**DOI:** 10.3390/ijerph16173016

**Published:** 2019-08-21

**Authors:** Antonio Ariza-Montes, Antonio L. Leal-Rodríguez, Jesús Ramírez-Sobrino, Horacio Molina-Sánchez

**Affiliations:** 1 Department of Management, Universidad Loyola Andalucía, 14004 Córdoba, Spain; 2 Facultad de Administración y Negocios, Universidad Autónoma de Chile, 425 Santiago, Chile; 3 Departamento de Administración de Empresas y Marketing, Universidad de Sevilla, 41018 Seville, Spain

**Keywords:** work engagement, authenticity, subjective wellbeing, faith-based organizations, partial least squares-PLS

## Abstract

Research in work and organizational psychology has paid little attention to religious workers, something certainly surprising as faith-based organizations play a key role in the welfare state of many countries. This research shows that religious workers in a Catholic order present a high degree of subjective wellbeing, both in terms of flourishing and satisfaction with life in general, and a positive balance of positive and negative feelings. More specifically, this study examines the relationship between authenticity and wellbeing amongst religious workers. Survey responses from 142 religious workers in Spain were analyzed using partial least squares path modelling. The results reveal that subjective wellbeing at work is positively related to authenticity. In addition, this relationship is mediated by their level of work engagement.

## 1. Introduction

It is widely acknowledged that innovations, technological advances, structural economic changes and social transformations are significantly modifying the nature and meaning of the labor market and are expected to predict the forthcoming shape of work relations. In this vein, the International Labour Organization (ILO) upholds an up-to-date and pertinent command—a mandate for the promotion of social justice [1] that points to four main investigation topics related with work and decent jobs. A direct outcome of such transformations within the workplace is the threatening of the employees’ perceived levels of health and wellbeing, which might be exposed to strong pressures to adapt or alter their own values to better fit the new organizational conditions, high levels of stress and even some kind of deteriorated working conditions (i.e., work stress, mobbing, bullying, etc.) [2,3]. Hence, it is becoming critical for organizations to promote decent jobs. This way, these challenges require a deeper scientific examination and understanding of the role of firms while boosting their employees’ level of subjective wellbeing.

With this regard, it must be noted that concern for the employees’ wellbeing has recently emerged as a relevant research topic within management literature [4], mainly due to its link with several personally- and organizationally-driven variables, such as satisfaction, organizational commitment, performance or happiness, among others i.e., [5,6,7].

Although the relationships between authenticity, wellbeing and work engagement are well established in different environments, to the best of our knowledge there is no empirical research that analyzes (1) the mediating role of work engagement in the authenticity–wellbeing relationship, and (2) less still in such a particular and slightly investigated group as is the one that occupies the attention of this study—consecrated members of a religious order. One point of high interest is the role of authenticity at work on work engagement [8,9,10,11,12,13]. In addition, authenticity at work and healthy psychological functioning display a reciprocal relationship, reinforcing mutuality as a virtuosity circle [14]. This research goes further into the role of work engagement in this authenticity–wellbeing relationship.

In this case, it is an order of Catholic inspiration and international scope, with a strong presence in Spain. At this point, we must stress the important research gap that exists on this issue. As pointed by [15], studies in work and organizational psychology have paid little attention to religious workers, something certainly surprising because they play an influential role in western societies. The astonishment increases if one takes into account that, in these organizations, feeling comfortable and acting consistently with one’s own personal beliefs, that is, to be and to act in an authentic way, might constitute a key driver of individual and collective commitment, thus, conditioning cohesion, the sense of belonging and, even, the degree of adherence to the project.

The management of feelings and emotions acquires special relevance in this group, since their life is devoted almost entirely to the service of others and therefore the separation between the working and personnel environment is practically non-existent. The happiness and wellbeing of these professionals are fed from their work with the poorest and most disadvantaged of society. In this context of almost absolute immersion in the workplace (religious workers live 24 h a day with their beneficiaries) it is possible that a mediating role of work engagement in the authenticity–wellbeing link might exist. Undoubtedly, employees’ engagement today constitutes a critical factor, as it is the most effective way of achieving organizational success [16]. As pointed out by [17], engaged workers are more likely to comply with their duties, a circumstance that is still more probable among religious workers, due to the reinforcement provided by the spiritual resources available to these people [18].

The information discussed above reveals a clear research gap to which this study aspires to contribute. Therefore, we have a double objective. First, does a direct relationship exist between authenticity at work and subjective wellbeing among religious workers? Second, does work engagement actually mediate the link between authenticity at work and subjective wellbeing?

## 2. Theoretical Framework

Subjective wellbeing is closely related with work engagement [19]. Under the job demand–resources model (JD–R), job characteristics are classified on job demands and job resources. They respectively start two processes: a health impairment path and a motivational process. The job demand–resources model proposes that job resources produce higher work engagement. Two of the utmost representatives authors of the job demand–resources model [20], identify work engagement scales as a tool for investigating employee wellbeing, with this suggestion deepening this theoretical framework. The main idea is that resources produce motivation and work engagement. These organizations mobilize job resources and among them, personal resources, such as optimism, self-efficacy and self-esteem. Vocational workers will experience positive outcomes in their life (optimism), and their faith contributes to feelings of self-efficacy and they can improve their feelings of self-esteem by participating in organizational demands. Ref [21] observed this positive influence of personal resources on work engagement and a reciprocal effect of work engagement on personal resources according to the conservation of resources theory. In faith-based organizations, this theoretical circle must be present, as employees need to feel their work is an authentic experience of their scale of values and beliefs.

### 2.1. Authenticity, Subjective Wellbeing and Work Engagement

Although from a philosophical approach authenticity has a long journey, the truth is that from an empirical perspective it constitutes a recent object of research. Starting from the initial studies of [22], different authors have emphasized the value of being authentic in different areas of personal and professional life. In fact, nowadays it is considered a fundamental topic within psychological research and other related fields [10].

Authenticity consists of acting coherently with feelings, beliefs, desires, preferences and personal values [23,24]. The possibility of acting in an authentic way produces positive effects, thus contributing to improvements in the organizational climate and reinforcing the organizational culture. However, the absence of authenticity translates into involuntary behaviors that bring about negative effects. According to [25], the three fundamental dimensions that integrate the authenticity construct are self-alienation, an authentic life and external influence acceptance. Although in practice, being completely authentic is certainly utopian, individuals will be more authentic whenever they are able to show high levels of authentic living in combination with low external influence acceptance and low self-alienation.

Against the scarce development of the authenticity concept, because of the enormous development and popularity lately attained by positive psychology, subjective wellbeing has attracted the interest of many academics [26]. This consideration has occurred despite the difficulty (or perhaps because of it) that involves dealing with a concept as complex and multifaceted as this. Classical philosophers already spoke of the “sum happiness”, understood as the only final value and as sufficient in itself, since once attained nothing more could be desired. Following [27], this highly vague and imprecise character has precisely motivated a progressive incorporation of more operational concepts within the psychological vocabulary, i.e., personal wellbeing and life satisfaction [28], and its antagonistic concept, ill-being [29].

More recently, [30] point out that the study of wellbeing has adopted two different perspectives of analyses, one hedonic (focused on happiness) and another eudemonic, related to the potential development of the individual. The hedonic approach would be directly linked to subjective wellbeing [31], although some authors such as [32] warn that we are facing a multidimensional phenomenon overlapping aspects inherent to both conceptions of wellbeing.

From this point of view, [33] developed an integrative proposal according to which subjective wellbeing would be integrated by three dimensions: (1) general satisfaction with life (hedonic paradigm); (2) positive and negative feelings (hedonic paradigm); (3) flourishing (hedonic and eudemonic paradigms), understood by [34] as a state of positive mental health.

The promotion of wellbeing is of interest to the members of organizations, but also for the owners. The organizations invest plenty of resources in the recruitment of people, a circumstance which, given the secularization of today’s society, is even more problematic and worrying in the case of the incorporation of new members into religious organizations. If these people experience poor health and wellbeing, they will have lower performance, will make worse decisions, will be less disposed to absenteeism [35] and, consequently, will decrease their contributions to the organization’s performance [36].

Without a doubt, the conditions and characteristics of work exert a multiplicative effect, both positive and negative on individuals’ wellbeing by affecting their behavior in the social, personal and familial context, and ultimately on competitiveness or efficiency [37]. For this reason, improving employees’ engagement is fundamental and of great interest to many social and professional scientists [38]. This view is shared by [39], who point out that the aspiration to involve, engage and obtain commitment from employees has been largely prioritized in the agenda of a selected portion of enlightened human resources managers.

When it comes to conceptualizing engagement there are two fundamental thinking streams. The first conception identifies work engagement with high levels of energy, participation and efficiency, namely the opposite poles of the three dimensions of burnout [40]. The alternative vision, although it accepts that engagement is the antithesis of burnout, poses it as a concept with its own entity.

Despite this controversy, different academics, such as [41] or [42], claim that work engagement is essential for organizations to achieve high performance and business success. From this perspective, [43] highlights that engagement is linked to essential aspects of human resource management: job rotation, productivity and profitability. Undoubtedly, employees’ work engagement translates into greater happiness and performance [9]. Workers committed to their work experience have a feeling of energetic and affective connection with the activities they perform, while perceiving themselves with sufficient capacities to cope with their work demands. From this point of view, work engagement would be a positive and persistent emotional affective state in employees, characterized by vigor, dedication and absorption [44]. Although in its origins it was recognized as a stable state related to work, it has subsequently been redefined, being considered today as a transitory experience that might fluctuate between different individuals [45].

### 2.2. The Authenticity–Wellbeing Link

Despite the fact that authenticity constitutes a nascent research topic [46], there is no doubt regarding its construct validity and robustness while predicting wellbeing [47]. Some authors actually consider authenticity to be the essence of wellbeing [22,25,48]. From this perspective, wellbeing is conceived as the extent to which individuals behave in an authentic manner under different situations and with distinct people. This way, while authenticity generates wellbeing, the lack of authenticity would lead to disorientation and dissatisfaction, as the individuals are pressured to act in opposition to their personal beliefs and values. Different studies have shown the association between authenticity and wellbeing. Without a doubt, there are more studies in line with the objective of this research, hence suggesting a positive tie between both concepts i.e., [47,49,50,51,52].

Previous studies have analyzed the influence of authenticity on subjective wellbeing in the workplace. Thus, using a sample of health sector workers in Australia, [53] found that authenticity leads to lower levels of tension, in a positive sense, and greater emotional wear, in a negative sense. Aside from this, [4] investigated within a sample of managers the mediator effect exerted by the degree of work significance in the authenticity– wellbeing link. Finally, using a hierarchical regression model in the German labor market, [10] demonstrated that self-alienation is the most decisive element of authenticity in the prediction of wellbeing.

**Figure 1:**
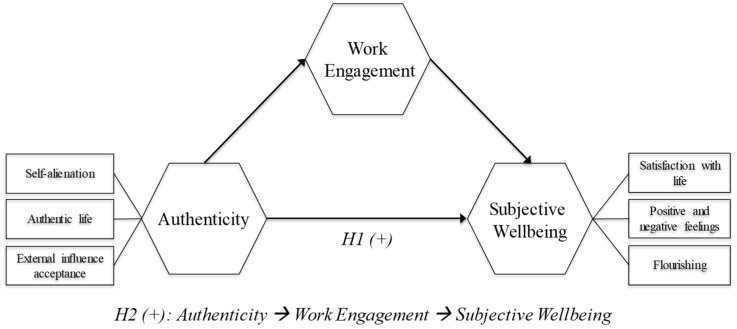
Research model and hypotheses.

In the specific perspective of religious organizations, it is likely that the ability to act and behave consistently with personal beliefs is decisive for the consecrated members. Consequently, we posit the next hypothesis (see Figure 1 for the model’s structure and variable interrelations):

**Hypothesis** **1.** *Authenticity is positively related to subjective wellbeing.*

### 2.3. The Mediating Role of Work Engagement in the Authenticity–Wellbeing Link

Reference [19] highlight the importance of work engagement in employees’ wellbeing and the fostering of positive behaviors at work. For this motive, the scientific literature has generated a series of studies that relate authenticity with work engagement and this last factor with subjective wellbeing.

Firstly, the authenticity–work engagement link has to do with individual awareness about oneself and the possibility of working in activities that allow for the implementation of the “true-self”, both at cognitive, personal and physical levels [54,55]. From this perspective, [9] sustain that those workers that act authentically at their work will be more motivated to comply with their duties, which leads to superior engagement. In this line, the study developed by [10] supports a positive authenticity–work engagement relationship. Their results, carried out with a sample of German employees, revealed that those employees who had the possibility of being themselves and work according to their own beliefs were more likely to experience positive results, including work engagement. Concretely, and contrarily to what these authors expected at first, they found that the “acceptance of external influences” dimension of the authenticity construct correlated positively and significantly with the “dedication” dimension of work engagement. Recently, [12] employed a structural equation model to analyze and support the link between authenticity and work engagement with a sample of German companies of the financial sector.

Secondly, another group of researchers have emphasized the link among work engagement and subjective wellbeing. The connection between these two constructs has to do with the fact that more-engaged workers will present more energy, feel greater enthusiasm for their work and are happy with the activity performed in their day-to-day life. Thus, [56] considers that it is more likely that these workers will perceive that their work positively affects their physical health and psychological wellbeing. In an analogous way, a study carried out by [57] linked work engagement with positive affects at the workplace, while suggesting that being engaged at work enriches the quality of life in other areas outside of the working environment [58]. Likewise, from a hedonic approach of subjective wellbeing, a study developed by [59] highlights that highly engaged workers develop positive emotions at work. Ref [60] arrived at the same conclusion with a sample of Spanish employees. These authors found that work engagement relates positively to personal happiness.

To the best of our knowledge, there is not a piece of empirical research that analyzes the mediating role of work engagement in the authenticity–subjective wellbeing link among members of religious organizations. Such a matter constitutes a gap of investigation that this article tries to cover. Consecrated members of religious organizations constitute undoubtedly a special collective. As indicated by [61], religious workers are a distinct occupational cohort within the helping professions who experience unique combinations of challenges, motivations, resources and demands. This situation implies that the effects of certain variables as the ones analyzed in this paper might present some special notes for this group of workers. The inclusion of the mediating role of work engagement in the authenticity–subjective wellbeing link is based on the fact that for the members integrating this collective, the best way of acting and being oneself (namely, remaining faithful to their beliefs and personal values) is through their daily work with poor and disadvantaged people, such as elderly people, unprotected minors and homeless people. This fact shows that the line that separates one’s work from one’s personal life is much more diffuse among consecrated people.

In general terms, the possibility of being oneself generates wellbeing on its own. However, in the case of religious individuals, we must bear in mind that what confers greater meaning to their lives is the “hitch” with their work, an activity to which they are devoted in body and soul, with all their vigor and full dedication that absorbs them completely. Their whole life is thus reduced to their work with the poorest. In this context of absolute dedication, the possibility of acting coherently with their beliefs and personal experiences materializes in an indirect way by means of work engagement with the beneficiaries of their work, which is what ultimately generates greater subjective wellbeing. Hence, we pose the following hypothesis (see Figure 1):

**Hypothesis** **2.** *The authenticity–subjective wellbeing relationship is mediated by work engagement.*

## 3. Methodology

### 3.1. Sample

An ad-hoc design questionnaire was circulated using Google Forms among the consecrated members of a Catholic organization. The respondents signed a consent form on this study. Data collection was carried out in the period April–May 2016. In this period, the target population amounted to 208 nuns. Participation was voluntary and completely anonymous. The response rate was 68.3% (142 valid questionnaires).

Table 1 displays the key constructs of this research. This table shows that the nuns exhibited a high authenticity degree, especially in the authentic life dimension (4.21 out of 5). Simultaneously, these individuals stated a high amount of subjective wellbeing: flourishing (4.5), satisfaction with life (4.0) and feelings (2.16). Finally, the surveyed nuns also seemed quite “engaged” in their jobs: dedication (4.50), vigor (4.33) and absorption (4.23).

As is visible in Table 1, there were significant correlations between the study variables. It can be seen that the authentic life dimension of the authenticity construct correlated positively and significantly with the three dimensions of subjective wellbeing (r _range_ = 0.213 to 0.543), something that only happens with the balance of positive–negative feelings in the case of self-alienation (r = 0.174) and acceptance of external influence (r = 0.241). Likewise, it was observed that an important correlation existed between having an authentic life with all the dimensions of work engagement (r _range_ = 0.299 to 0.410). Finally, there was an intense positive and meaningful correlation between vigor, dedication and absorption, on the one hand, and self-alienation, authentic living and external influence acceptance (r _range_ = 0.361 to 0.685) on the other.

### 3.2. Measures

Authenticity is measured with the IAM (Individual Authenticity Measure at Work) scale developed by [10]. This instrument comprehends three different constructs: authentic life (i.e., “At work, I always stand by what I believe in”), external influence acceptance (i.e., “At work, I feel the need to do what others expect me to do”) and self-alienation (i.e., “At work, I feel out of touch with the ‘real me’”). The reliability estimates in this study for the three dimensions were 0.691 (authentic life), 0.722 (self-alienation) and 0.755 (external influences acceptance).

A scale developed by [33] was used to measure the three dimensions of subjective wellbeing: Satisfaction with Life Scale (i.e., “If I could live my life over, I would change almost nothing”), Scale of Positive and Negative Experience, that comprises a group of positive and negative feelings and the Flourishing Scale of [62]. Some items of this scale include “At work I am competent and able to carry out the activities that are important to me” or “My work enables me being a good person and living a good life”. The Cronbach’s alpha for the three dimensions of this scale were 0.824 (satisfaction with life), 0.728 (positive and negative experience) and 0.855 (flourishing).

The Utrecht’s Scale of Work Engagement, developed by [63], was used to measure work engagement. This instrument comprised three constructs: vigor (i.e., “At my work, I feel bursting with energy”), absorption (i.e., “I get carried away when I’m working”) and dedication (i.e., “I am proud on the work that I do”). Our research obtained an alpha coefficient of 0.696 (absorption), 0.791 (vigor) and 0.824 (dedication).

The conceptual variables that shape our research are modeled as composite constructs. A composite construct is shaped as a linear combination of its own manifest variables or dimensions [64]. Thus, deleting one or more indicators frequently modifies the meaning of the construct [65] as they embody distinct facets where high correlations among indicators might be expected, yet they are not mandatory [66]. The decision to model the conceptual variables as composites rather than using a common factor model derives from the fact that such variables are artifacts or design constructs instead of behavioral constructs.

### 3.3. Data Analysis

This paper employs partial least squares (PLS-SEM) to empirically examine the model and structural relationships proposed. PLS is a variance-based structural equation modeling (VB-SEM) approach [67] that enables the simultaneous appraisal of the measurement model (i.e., assessing the reliability and validity of the measures of conceptual variables) and of the structural model (i.e., analyzing the structural links hypothesized between the constructs comprised at the model) [68]. PLS-SEM was selected principally because the latent variables that form the model are measured as composites, namely, human-made instruments or artifacts that are conceptually supported and frequently crafted by individuals (i.e., managerial and staff job-holders within organizations)—for instance, managerial procedures or techniques, individual or organizational innovativeness, or information systems [65,66]. The usage of PLS-SEM with composite measurement models has been equally endorsed at a theoretical level [64,69] and an empirical level [70,71]. Given that such design constructs or artifacts are formed out of a set of basic elements or parts that are combined to shape a new entity, [66] suggests that they should be modeled as composites. Thus, in composite measurement models, constructs are represented as weighted linear combinations of its indicators [72], where indicators are not assumed to be causing nor reflecting the construct, but forming or composing it [66]. This way, the composite model relaxes the strict assumption imposed in the common factor model relative to the fact that any covariation between the indicators is explained by a common factor. Social science research is a scientific discipline that studies artifacts as well as behavioral phenomena. Thus, empirical research demands techniques that enable modeling design as well as behavioral constructs. Variance-based structural equation modeling (VB-SEM) and particularly PLS-SEM is presented as a technique that enables the simultaneous assessment of models that comprise both type of constructs [66]. Secondly, accordingly with [73], this study uses PLS-SEM since it employs latent variable scores in a successive analysis for modeling superordinate (second order or multidimensional) constructs, by using the two-stage approach [74]. The three latent variables shaping the research model were measured using Mode A, both at the first-order (dimension) and second-order construct levels. This mode uses correlation weights, and it is suitable for the estimation of standardized regression coefficients in small to medium sized samples, and when indicators are correlated among each other [70]. Third, this study had the purpose of predicting the dependent variables rather than following confirmation purposes [75]. The focus on prediction over confirmation purposes is a pivotal motive to use PLS-SEM instead of covariance-based SEM (CB-SEM) techniques. In CB-SEM, the aim is to minimize the discrepancy between the data and the model, namely, attaining a satisfactory fit between both matrixes. On the contrary, in PLS-SEM the goal is to explore the sign and significance of the linkages hypothesized, as well as to maximize the explained variance by means of the coefficient of determination (R2). This is the goal in this paper, to explore whether authenticity and work engagement drive or predict subjective wellbeing within the particular context of assessment, and to test whether there is a mediation effect. Fourth, in line with [67], we considered that the model is complex because of the modeling of the variables as second-order (multidimensional or superordinate) constructs shaped by various dimensions or facets, and also because of the nature of the relationships hypothesized (mediation or indirect link). These authors believe these characteristics to be idiosyncratic of model complexity and endorse the usage of PLS-SEM in these cases. Finally, this study employed the SmartPLS 3.2.8 software [76].

## 4. Results

The evaluation of PLS-SEM models comprises three steps: (i) assessing global model fit, (ii) validating the reliability and validity of the measurement model and (iii) weighing the sign and significance of the structural relationships among constructs.

### 4.1. Assessing Global Model Fit

Reference [65] suggest the evaluation of global model fit as a preliminary step in PLS-SEM analyses. When there is no fitting between the data and the model, this implies that the data includes additional information that the model lacks. Hence, we employed ADANCO 2.0.1 in order to execute a set of bootstrap-based model fit checks [77]. Concretely, we relied on the use of three tests: (i) the standardized root mean squared residual (SRMR), (ii) the unweighted least squares discrepancy (dULS) and (iii) the geodesic discrepancy (dG). According to [66], for an accurate model, the values of these three tests should not surpass the bootstrap-based 95% (HI95) or 99% (HI99) percentiles. Model fit results show that the three tests are well under HI95 and HI99 (see Table 2). In addition, the SRMR was employed as an estimated model fit statistic that reveals whether the divergence between the conceptual model and the empirical correlation matrix is high or not [78]. In this vein, [65] suggest a critical level of 0.08 to attain satisfactory model fit in PLS-SEM. In our model, SRMR attains an acceptable value of 0.075 (see Table 2).

### 4.2. Measurement Model

Given that all the multidimensional constructs were artifacts (design constructs) estimated in Mode A, it was likely that the manifest variables (indicators or dimensions) employed to measure the composite constructs within the model would be correlated [64]. Hence, it permitted the application of conventional tests for measuring internal consistency, reliability and validity [79]. All the manifest variables had generally outer loadings above the 0.707 cutoff and only some of them were marginally below this threshold. Hence, our choice was to keep them to maintain the content validity of the measurement scale. Consequently, individual item reliability was considered satisfactory in this study (Table 3). Aside from this, all the latent variables complied with the requisite of construct-level reliability, since the values observed for the Cronbach’s alpha, Dijkstra–Henseler’s indicator (Rho_A) and composite reliabilities were over the 0.7 cutoff (Table 3). Moreover, these latent variables attained convergent validity, given that their average variance-extracted (AVE) values surpassed the 0.5 cutoff (Table 3). Finally, Table 4 disclosed that the three latent variables reached discriminant validity accordingly with the Fornell–Larcker and the Heterotrait–Monotrait (HTMT) approaches [79]. This means that the multidimensional constructs significantly differ from each other.

### 4.3. Structural Model

**Figure 2:**
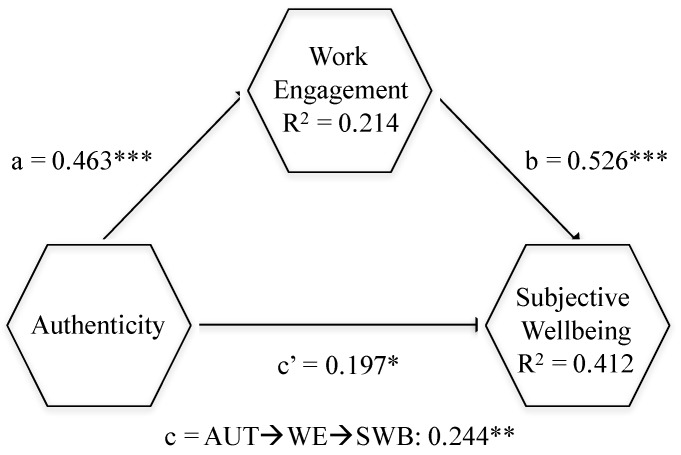
Structural model results. *** *p* < 0.001; ** *p* < 0.01; * *p* < 0.05.

Coherently with [81], a 5000 resample bootstrapping procedure was computed to engender *t*-statistics, *p*-values, standard errors and 95% BCCI (bias corrected confidence intervals) that allowed the assessment of the significance of the links encompassed within the research model (Table 5). The main criterion employed to assess the amount of explained variance of the dependent constructs was the coefficient of determination (R2 coefficient). Thus, the results gathered in Table 5 endorsed the structural model that this paper posits, exhibiting that it offered satisfactory predictive power for the endogenous constructs. Aside from this, all the direct effects hypothesized in this model were shown to be positive and significant (Table 5). This study also found support for the existence of a mediation (indirect) effect of work engagement on the authenticity–subjective wellbeing link (Table 6). It is important to highlight that the direct AUT-SWB link did not become non-significant once the mediator variable (WE) was introduced in the model. On the contrary, this direct link remained positive and significant. This implies that empirical results sustain the existence of a partial mediation rather than full mediation. Figure 2 summarizes the main structural model results.

### 4.4. Importance–Performance Map Analysis

This section intends to present additional findings that may provide more insight from the PLS results. Thus, this section enlightens PLS results by means of the importance–performance map analysis (IPMA), a helpful analysis approach in PLS-SEM provided by the SmartPLS 3.2.8 package [82]. This analysis expands the standard PLS outcomes through the addition of a dimension that considers the average latent variable scores values. Concretely, this procedure contrasts the total effects, embodying the exogenous constructs’ relevance in determining a certain target construct, with their average values of latent variable scores representing their performance [82]. Hence, this technique is aimed at identifying antecedents or drivers that are important to determine the target construct (i.e., those with robust total effects), but reveal low performance also (i.e., low latent variable scores values).

**Figure 3:**
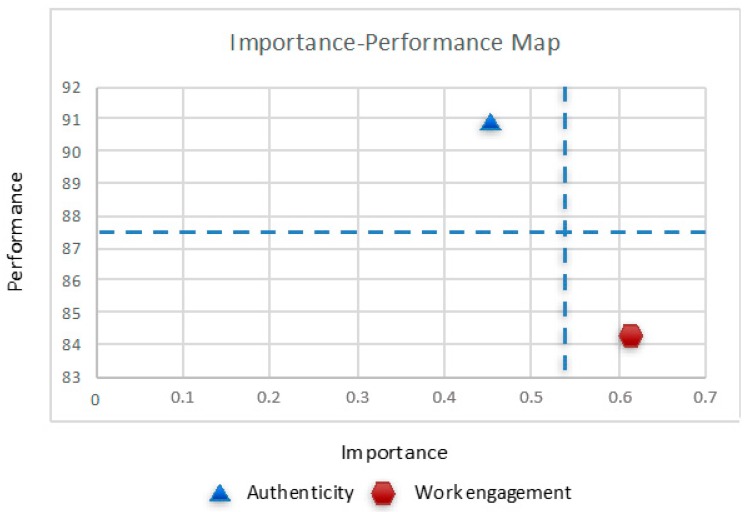
Importance–performance map.

The importance and performance values of SWB’s antecedent constructs (i.e., authenticity and work engagement) enable the building of the importance–performance map of SWB. Table 7 contains the importance and performance values for the antecedent constructs and mean values. Subsequently, these data can be translated into a scatter plot, which permits the creation of an importance–performance map, as exhibited in Figure 3. The *x*-axis embodies the relevance of authenticity and work engagement while explaining the dependent construct (SWB), whereas the *y*-axis portrays the performance of authenticity and work engagement in terms of their average rescaled latent variable scores. To achieve a more precise orientation, two auxiliary lines are drawn in the importance–performance map, shaping the mean values for the importance and performance dimensions (i.e., a vertical and a horizontal line, respectively) (Figure 3). In our model, our results reveal an average importance of 0.536 and an average performance of 87.455 (Table 7). The two auxiliary lines split the map into four quadrants that depict the values for importance and performance dimensions above and below the mean values. Generally, while conducting IPMA, the variables placed in the lower-right quadrant (i.e., scoring below average in terms of performance and above average in terms of importance) are the most interesting and the ones whose assessment should be prioritized. In second place, the study of the variables placed at the higher-right, lower-left and, finally, the higher-left quadrants should be emphasized. Thus, IPMA may offer advice regarding what constructs should be prioritized [82].

In our paper, the IPMA technique provided interesting results. Since the WE construct was paced at the lower-right quadrant, this implies that it scored over the average in terms of importance and under the average in terms of performance. Thus, work engagement is the aspect that should be prioritized. This result is in line with the mediation effect hypothesized and supported by PLS analysis that entails that authenticity is a significant driver of subjective wellbeing, but only to the extent that it leads to work engagement. In other words, only when authentic behavior contributes to raise the individuals’ levels of work engagement will it subsequently lead to increasing their perceived subjective wellbeing.

## 5. Discussion

Employee’s well-being in the context of faith-based organizations is an issue particularly critical to the extent that different authors, such as [83] or [84], have empirically tested the influence of religious variables on wellbeing. From this premise, and in order to achieve healthy working environments, the human resource managers of these organizations should bear in mind that the wellbeing of their members is a nuclear motivating force. On the contrary, on many occasions it is a careless aspect, perhaps because in the case of consecrated members of religious orders wellbeing and motivation are presumed, in some cases erroneously, such as value in soldiers. This research shows that religious workers in a Catholic order present a high degree of subjective wellbeing, both in terms of flourishing and satisfaction with life in general, as well as a positive balance of positive and negative feelings. More specifically, this study examines the relationship between authenticity and wellbeing amongst religious workers. The results reveal that subjective wellbeing at work is positively related to authenticity. In addition, this relationship is mediated by their level of work engagement.

At this point, we must stress the important research gap that exists on this issue. As pointed by [15], studies in work and organizational psychology have paid little attention to religious workers. It is possible that the scarcity of empirical research is motivated by the difficulty that exists to accede to this group of people, both because of their small number if compared to other forms of employees, as well as by the prudence that they usually adopt while collaborating with this type of research because of their life choice, one that is more oriented to spiritual retreat than public exposition. Therefore, we believe that it constitutes a value in itself to have gathered a sample of 142 religious workers (most of them nuns) in Spain.

This study contributes to the wellbeing literature in the context of faith-driven organizations. Firstly, the PLS analysis concludes that religious workers’ wellbeing is positively impacted by authenticity perception. This means that employees feel more authentic if they carry out their job in a work environment, which is consistent with their core “authentic” self. In consequence, those that might show themselves as they actually are and are not forced to dissimulate or adopt hypocritical behaviors will experience a higher level of subjective wellbeing in its three dimensions: satisfaction with life, flourishing and balance of positive and negative feelings. In sum, this means that to increase their level of wellbeing, they must search for an authentic and fully realized life. Their conditions as “owners” of the institution must make this way of living easier [52]. This evidence, that confirms the approach of our first research hypothesis, is consistent with the results discovered by other researchers, such as [4,11,12], among others.

Secondly, work engagement is key for the development of subjective wellbeing of religious employees, a role that manifests both directly and, more notably, in the relationship between authenticity and subjective wellbeing, as the findings also revealed that work engagement was a significant mediator of this. This finding is meaningful, since engaged employees work with extraordinary endeavor are more engaged and are more likely to go beyond the expectations and work requirements [85]. Furthermore, this circumstance seems especially significant among religious workers, given the fragile line that separates their personal lives from their professional lives, to the point of erasing the borders that usually exist between public and private life [86]. To work and to be delivered in body and soul to others constitutes an aspiration for most of the religious workers, a nuclear element of their vital expectations. The existence of a positive direct impact of work engagement on subjective wellbeing is in line with prior studies that addressed this issue, although all of them were carried out in contexts distinct to this study [17,60,87,88,89,90,91,92,93,94,95]. According to [96], work engagement among religious leaders is an under-investigated topic. Therefore, this study attempts to cover a scarcely explored research gap.

Lastly, this study revealed that authenticity leads to engagement which, in succession, contributes to happiness. These findings highlight the positive tie existing between authenticity and subjective wellbeing, through vigor, dedication and absorption in a captivating and gratifying workplace context. However, the same work environment might also be often labelled as frustrating and discouraging. This involves that the effect exerted by authenticity on wellbeing is more intense when it takes place across work engagement. The explanation for this fact may be motivated again by the importance that work acquires in this group of people, in such a way that the possibility of leading an authentic life at the service of the poorest, as promulgated by the charism of the Order, would move to daily work with the elderly, disabled, children, prostitutes, etc., which would, in turn, induce them a greater degree of subjective wellbeing. Consecrated members of this type of religious order consider that doing good through their dedication to God is not to be realized through contemplative life, but through the dedication to work—its main source of satisfaction and wellbeing. Without a doubt, considering a job as “a calling” will allow people to work more [97]. In fact, a longitudinal study developed by [98] showed that the link with a spiritual call generated more work engagement among consecrated religious workers than amongst non-religious ones. According to these authors, spiritual resources engage these individuals to work by equipping them with greater meaning and by increasing their sense of effectiveness in their performance. If all this is coupled with the reinforcement of spiritual beliefs among religious workers [18], the consequence can be no other than an increase in individual wellbeing.

In conclusion, authenticity of religious workers in the workplace is significantly related to their wellbeing. It seems that work engagement could be a determining factor for the link between authenticity and subjective wellbeing at work. The current research is original because it empirically explores authenticity in the context of faith-based organizations. In addition, it assessed the positive linkage between authenticity and religious workers’ wellbeing at work. In sum, this study proposes to go further on the knowledge about authenticity and wellbeing among religious workers through a greater level of engagement at work. Furthermore, the outcomes derived from importance–performance map analysis (IPMA) revealed that this organization should make an effort to improve its performance as for the fostering of their employees’ work engagement.

## 6. Limitations and Future Research

Like most studies in social science research, this work presents some limitations that must be considered while interpreting the results obtained. Thus, the limitations inherent in cross-sectional research were found in that all data were collected through self-perception, which can lead to bias in the information obtained. Moreover, the cross-sectional design caused predictive relationships found among the constructs to not warrant strong causal inferences. Another important aspect to take into account is the effect of social desirability in a twofold sense: on the one hand, because of the very topical approach to research (authenticity, work engagement and subjective wellbeing); on the other hand, because of the idiosyncrasy of the collective analyzed. Finally, we should be cautious when it comes to generalizing these results to other types of religious workers. This restriction is manifested both at the geographical level (the study focused on Spain) and ideological (the study focused on a Catholic religious order). In future research, we propose to extend the sampling to different geographical and cultural contexts, in order to include individuals holding a more diverse background. Additionally, to have a comparative sample of non-religious employees would be an interesting idea for future research. Finally, another interesting future research project could be to test the issue of lack of authenticity in religious workers and its effects on their wellbeing and work engagement.

## Figures and Tables

**Table 1:** Descriptive statistics and inter-correlations for the study variables.

Variable	*M*	*SD*	Range	1	2	3	4	5	6	7	8	9
1. Self alienation	3.75	1.14	1–5	1								
2. Authentic living	4.21	0.73	1–5	0.077	1							
3. Accep. ext. infl.	3.43	1.07	1–5	0.558 **	0.059	1						
4. Life satisfaction	4.00	0.83	1–5	−0.065	0.327 **	−0.063	1					
5. Feelings +/-	2.16	1.25	(†)	0.174 *	0.213 *	0.241 **	0.396 **	1				
6. Flourishing	4.50	0.65	1–5	0.113	0.543 **	0.114	0.479 **	0.390 **	1			
7. Vigour	4.33	0.75	1–5	0.119	0.356 **	0.171 *	0.412 **	0.515 **	0.590 **	1		
8. Dedication	4.50	0.72	1–5	0.133	0.410 **	0.122	0.456 **	0.418 **	0.685 **	0.721 **	1	
9. Absortion	4.23	0.79	1–5	−0.026	0.299 **	−0.050	0.479 **	0.361 **	0.537 **	0.532 **	0.639 **	1

(†) This variable is calculated as the difference between the positive and negative feelings (on a scale of 1 to 5) experienced by the religious members. * *p* < 0.05; ** *p* < 0.01

**Table 2:** Tests of model fit.

	Value	HI95	HI99
SRMR	0.075	0.197	0.212
d_ULS_	6.013	41.038	47.617
d_G_	19.246	66.254	68.983

Notes: SRMR: standardized root mean squared residual; dULS: unweighted least squares discrepancy; dG: geodesic discrepancy; HI95: bootstrap-based 95% percentile; HI99: bootstrap-based 99% percentile.

**Table 3:** Measurement model: loadings, construct reliability and convergent validity.

Construct/Dimension	Loading	Cronbach’s Alpha	rho_A	Composite Reliability	AVE
**Authenticity** (Composite, Mode A)		0.783	0.788	0.874	0.699
Self-alienation	0.886				
Authentic life	0.629				
External influence acceptance	0.711				
**Work engagement** (Composite, Mode A)		0.835	0.870	0.901	0.752
Absorption	0.810				
Dedication	0.914				
Vigour	0.878				
**Subjective Wellbeing** (Composite, Mode A)		0.772	0.735	0.810	0.589
Satisfaction with life	0.826				
Positive and negative feelings	0.839				
Flourishing	0.463				

Note: Rho_A: Dijkstra-Henseler’s indicator; AVE: average variance extracted.

**Table 4:** Measurement model: discriminant validity.

**Fornell-Larcker Criterion**			
	**Authenticity**	**Subjective Wellbeing**	**Work Engagement**
Authenticity	0.657		
Subjective Wellbeing	0.440	0.681	
Work Engagement	0.463	0.617	0.869
**Heterotrait-Monotrait Ratio (HTMT)**			
	**Authenticity**	**Subjective Wellbeing**	**Work Engagement**
Authenticity			
Subjective Wellbeing	0.713		
Work Engagement	0.539	0.814	

Note: Fornell-Larcker criterion: Diagonal elements (italics) are the square root of the variance shared between the constructs and their measures (AVE). For discriminant validity, diagonal elements should be larger than off-diagonal elements. Off-diagonal elements are the correlations among the constructs. Heterotrait-Monotrait Ratio (HTMT) criterion should be under the threshold of 0.85 [80].

**Table 5:** Effects on endogenous variables.

Endogenous Variable	Direct Effect	*p*-Value	*t*-Value	95% BCCI	Support
**Work Engagement (R^2^ = 0.214)**					
Authenticity (+)	0.463 ***	0.000	3.826	[0.220; 0.670]	Yes
**Subjective Wellbeing (R^2^ = 0.412)**					
Authenticity (+)	0.197 *	0.053	1.936	[0.004; 0.424]	Yes
Work Engagement (+)	0.526 ***	0.000	5.435	[0.346; 751]	Yes

Note: Bootstrapping 95% confidence interval bias corrected in square brackets (based on *n* = 5000 subsamples). *** *p* < 0.001; ** *p* < 0.01; * *p* < 0.05 (based on *t* (4999), one-tailed test). *t* (0.05. 4999) = 1.645; *t* (0.01. 4999) = 2.327; *t* (0.001. 4999)= 3.092; ns = not significant, BCCI = bias corrected confidence intervals.

**Table 6:** Summary of mediating effect tests.

Total Effect of AUT on SWB				Direct Effect of AUT on SWB				Indirect Effect of AUT on SWB Via (WE)			
Path Coefficient	*p*-Value	*t*-Value	95% BCCI	Path Coefficient	*p*-Value	*t*-Value	95% BCCI	Path Coefficient	*p*-Value	*t*-Value	95% BCCI
0.440 ***	0.001	3.495	[0.234; 0.701]	0.197 *	0.053	1.936	[0.004; 0.424]	0.244 **	0.006	2.756	[0.121; 0.447]

Note: Bootstrapping 95% confidence interval bias corrected in square brackets (based on *n* = 5000 subsamples). *** *p* < 0.001; ** *p* < 0.01; * *p* < 0.05 (based on *t* (4999), one-tailed test). *t* (0.05. 4999) = 1.645; *t* (0.01. 4999) = 2.327; *t* (0.001. 4999)= 3.092; ns = not significant, WE = mediator variable.

**Table 7:** Data of the importance-performance map for SWB.

Antecedent Construct	Importance	Performance
**Authenticity**	**0.454**	**90.752**
Work engagement	0.617	84.158
Mean value	0.536	87.455
